# Moonlighting Proteins in the Fuzzy Logic of Cellular Metabolism

**DOI:** 10.3390/molecules25153440

**Published:** 2020-07-29

**Authors:** Haipeng Liu, Constance J. Jeffery

**Affiliations:** 1 Center for Biomolecular Sciences, College of Pharmacy, University of Illinois at Chicago, 900 South Ashland Avenue, Chicago, IL 60607, USA; hliu47@uic.edu; 2 Department of Biological Sciences, University of Illinois at Chicago, 900 South Ashland Avenue, Chicago, IL 60607, USA

**Keywords:** moonlighting proteins, fuzzy logic, intrinsically disordered proteins, metamorphic proteins, morpheeins

## Abstract

The numerous interconnected biochemical pathways that make up the metabolism of a living cell comprise a fuzzy logic system because of its high level of complexity and our inability to fully understand, predict, and model the many activities, how they interact, and their regulation. Each cell contains thousands of proteins with changing levels of expression, levels of activity, and patterns of interactions. Adding more layers of complexity is the number of proteins that have multiple functions. Moonlighting proteins include a wide variety of proteins where two or more functions are performed by one polypeptide chain. In this article, we discuss examples of proteins with variable functions that contribute to the fuzziness of cellular metabolism.

## 1. Introduction

**Figure 1:**
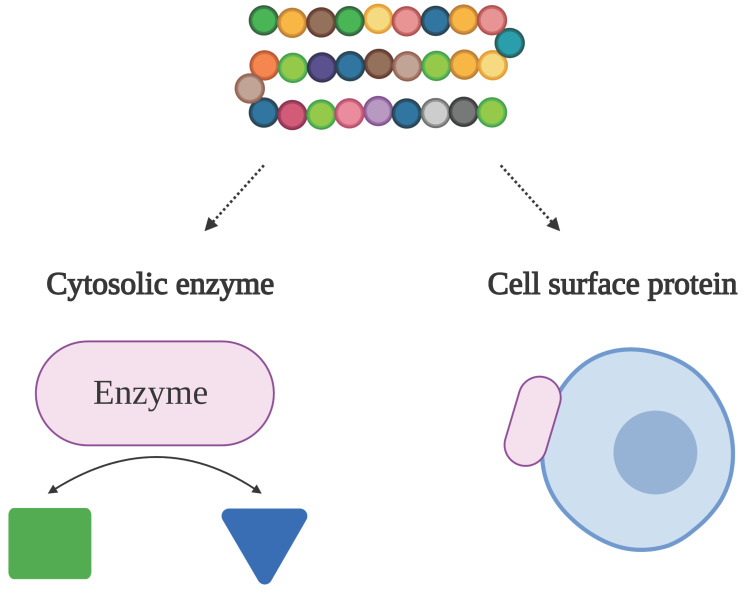
In a moonlighting protein (purple oval), more than one physiologically relevant biochemical or biophysical function is performed by a single polypeptide chain. Note: This figure was “Created with BioRender.com”.

Fuzzy logic systems include variables that can be any real number between 0 and 1 instead of being limited to the Boolean logic variables of only 0 and 1. This enables expression of complexity, uncertainty, and imprecision. In general, the vast interconnected biochemical pathways that make up the metabolism of a living cell can appear fuzzy because they are complex and hard to predict, and we have incomplete and not yet accurate knowledge. A single cell contains thousands of proteins performing a wide variety of activities, and the proteins have complex and constantly changing levels of expression, levels of activity, and patterns of interactions with other proteins and other molecules. Adding even more layers of complexity is the ability of many proteins, called moonlighting proteins, to perform more than one function. Moonlighting proteins are proteins in which one polypeptide chain performs more than one physiologically relevant biochemical or biophysical function [1,2,3] (Figure 1). The MoonProt Database (www.moonlightingproteins.org) contains annotations for over 300 experimentally confirmed moonlighting proteins, of which about 130 proteins are from human [4,5]. Although the mechanisms by which one protein performs two different functions are not always understood, it is clear that the function (or functions) performed at any specific time can be affected by multiple factors, and sometimes combinations of factors, including targeting to different cellular compartments, changes in the intracellular concentration of ligands, and changes in environmental conditions. In this paper, we describe examples of moonlighting proteins and some of the mechanisms by which they change function. These examples help illustrate and complement the ideas in this collection of papers on the topic of “The Fuzziness in Molecular Supramolecular, and Systems Chemistry”, where Gentili presents the “Fuzziness of the Molecular World” and describes natural information systems that involve fuzzy logic in large part due to proteins having multiple features and functions that vary in a context-dependent manner [6]. In addition, the paper by Fuxreiter describes using fuzzy set theory in a quantitative framework for describing the relationships between changing protein structures, interactions, and functions under changing, and somewhat unknown or unpredictable, cellular conditions [7].

## 2. Examples of Moonlighting Proteins and Factors that Affect Function

### 2.1. Cellular Localization

The most often observed subclass of moonlighting protein includes proteins that perform different functions in different cellular localizations. Over 100 enzymes and chaperones that catalyze reactions in the cytosol can be secreted and act as cytokines that modify the host’s immune system or become bound to the cell membrane where they serve as cell surface receptors, and in some cases these second functions contribute to virulence [8,9,10,11].

**Figure 2:**
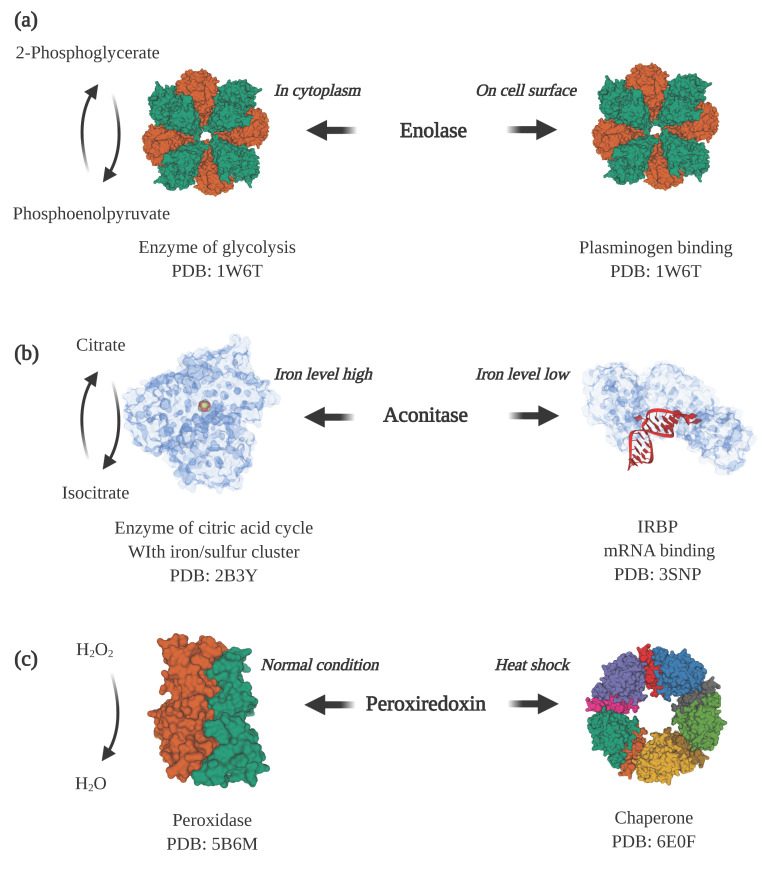
Examples of moonlighting proteins. (**a**) Enolase is a cytosolic enzyme in glycolysis and also a plasminogen receptor when displayed on the cell surface (PDB ID: 1W6T [31]). (**b**) Aconitase is an enzyme in the citric acid cycle when it contains an iron/sulfur cluster bound in the active site of the protein (PDB ID: 2B3Y [32]). When the cellular iron level decreases and the iron/sulfur cluster disassociates, aconitase undergoes a large conformational change that enables it to bind to iron-responsive elements in mRNA (PDB ID: 3SNP [33]) to promote the expression of proteins involved in iron uptake. (**c**) Under normal cellular conditions, peroxiredoxin is predominantly a dimer (PDB ID: 5B6M [34]) that functions as a peroxidase that converts hydrogen peroxide to water. Under heat shock or oxidative stress, it converts to a higher molecular weight form, a decamer, that acts as a molecular chaperone that assists with protein folding (PDB ID: 6E0F [35]). Note: This figure was “Created with BioRender.com”, and the visualizations of the protein structures were created with Mol* [36] on the RCSB PDB website (rcsb.org) [37,38].

Enolase is one of these intracellular/surface moonlighting proteins in many species, including eukaryotes as well as prokaryotes. Inside the cell, it catalyzes the conversion of 2-phosphoglycerate to phosphoenolpyruvate in glycolysis. When displayed on the cell surface, it binds to host proteins (Figure 2a). The enolases from *Aeromonas hydrophila*, *Bacillus anthracis*, *Neisseria meningitidis*, *Streptococcus pneumoniae*, *Trichomoniasis vaginalis* and *Lactobacillus crispatus* can bind to host plasminogen [12,13,14,15,16,17]. The binding of plasminogen plays an important role in invasion of host tissues because, once bound to the cell surface receptor, the plasminogen becomes converted to the active protease, plasmin, which can aid in breaking down host extracellular matrix and invasion of tissues [18,19]. In some species, surface-located enolase and other intracellular/surface moonlighting proteins bind to other host proteins for colonization or for modulating the host immune system. *Streptococcus suis* enolase can also bind to host fibronectin, and *Staphylococcus aureus* enolase exhibits laminin binding activity [20,21].

Glyceraldehyde 3-phosphate dehydrogenase (GAPDH) is another commonly found intracellular/surface moonlighting protein. It catalyzes the conversion of glyceraldehyde 3-phosphate to glycerate 1,3-bisphosphate in glycolysis in the cytoplasm. Some commensal bacteria that colonize the human gut use GAPDH on the cell surface to bind to host mucin and enable colonization of the gut [22,23]. When expressed on the cell surface, *Streptococcus pyogenes* GADPH can bind to plasminogen and fibronectin, and can also function as a ADP-ribosylating enzyme and assist with neutrophil evasion [24,25,26,27]. In addition, *Streptococcus agalactiae* GADPH can act as a modulator of the host’s immune system [28].

As the bacterial HSP70 (heat shock protein 70), DnaK is abundantly expressed in the cytosol as a stress-inducible chaperone. *Mycobacterium tuberculosis* DnaK can be displayed on the cell surface to bind to plasminogen, and it can also bind to CD40 (cluster of differentiation 40) to stimulate the synthesis of monocyte chemokines and the maturation of dendritic cells [29,30].

### 2.2. Interactions with Other Proteins and Molecules

Changes in cellular concentration of substrates or other ligands can serve as a trigger for changing protein functions. Aconitase is an enzyme in the citric acid cycle that contains an iron–sulfur cluster in the active site (Figure 2b). When the intracellular concentration of iron is high, the iron–sulfur cluster in the active site enables the isomerization of citrate to isocitrate [39]. In contrast, when the cellular level of iron is low, the iron–sulfur cluster is lost, and the enzyme changes conformation to expose an RNA binding surface and becomes an iron-responsive element binding protein (IRBP). As an IRBP, it binds to Iron Responsive Element (IRE) sequence motifs in mRNA, leading to increased translation of proteins involved in cellular iron uptake [40,41]. Another example is BirA, which performs functions as an enzyme and a transcription repressor in the biotin regulatory system [42]. The enzyme’s function is determined by the cellular need for biotin. When the need is high, when the cells are growing rapidly, BirA functions as a biotin–[acetyl–CoA-carboxylase] ligase that transfers a biotinyl moiety to the biotin carboxyl carrier protein subunit of acetyl–CoA carboxylase. When the demand decreases, under slower growth rates, BirA binds to DNA and functions as a biotin–operon repressor to inhibit the production of biotin [43].

As with aconitase and BirA, the different functions of many moonlighting proteins require interactions with different proteins, multiprotein complexes, DNA, RNA and other macromolecules. Many ribosomal proteins are moonlighting proteins, such as ribosomal proteins S3, S13, S14, L2, L4, L5, L7, L11, L13a, L23 and L26 [44,45,46,47,48,49,50,51,52,53,54]. These proteins have a second function when they dissociate from the ribosome and interact with other molecules. For example, the ribosomal protein S3 is a component of the 40S subunit of the ribosome, which is located in the cytoplasm. When S3 dissociates from the ribosome, it can enter the nucleus and act as a deoxyribonuclease (DNase) that cleaves apurinic/apyrimidinic sites during DNA repair [55]. Like the cytosolic aconitase mentioned above, mitochondrial aconitase acts as an enzyme in the citric acid cycle to catalyze the isomerization of citrate to isocitrate. However, instead of a function in translation, mitochondrial aconitase plays an important role in the maintenance of mitochondrial DNA that is independent of its catalytic role in the citric acid cycle [56].

### 2.3. Environmental Stress

Another common factor for moonlighting proteins to switch functions is environmental stresses. One example is peroxiredoxin, which in many species changes function from an enzyme to a protein-folding chaperone in response to oxidative stress or heat shock [57] (Figure 2c). In a lower molecular weight form, peroxiredoxin acts as a peroxidase that reduces hydrogen peroxide to water. However, under stress conditions, peroxiredoxin undergoes a shift to a higher molecular weight homo-oligomeric complex, comprised of five dimers connected by hydrophobic interactions [35]. The high molecular weight complex is a molecular chaperone that helps with folding and stabilizing proteins disrupted by the cell stress conditions.

Another example is the DegP protease, which also has a temperature-dependent change of function, where it transitions between a protease and a molecular chaperone. Under low temperature conditions, DegP functions as a molecular chaperone with the proteolytic site inactivated. When temperatures increase, the proteolytic site is activated and DegP can catalyze protein degradation [58].

### 2.4. Changes in Protein Structure

Changes in cellular localization, interaction partners, and environmental conditions can trigger a change in protein function with little or no change in protein structure. For example, in some moonlighting enzymes that are also transcription factors, ligand binding can turn on the transcription factor function by causing a relatively small change in overall structure that increases its DNA binding affinity [59]. Other moonlighting proteins undergo a large conformational change, such as cellular aconitase becoming IRBP, which involves a large relative movement of several domains within a protein subunit to uncover a previously buried mRNA binding surface. The peroxiredoxins described above undergo changes in quaternary structure to become chaperones.

In general, many moonlighting proteins undergo changes in structure, which can range from small movements of surface loops to the more drastic change in tertiary or quaternary structure observed in intrinsically disordered proteins (IDPs), metamorphic proteins, or morpheeins.

Intrinsically disordered proteins have a region or subunit that is unfolded and can in some cases enable a switch from unfolded to multiple folded structures that enable interactions with different proteins. Metamorphic proteins contain a domain or subunit that folds into more than one stable structure. Morpheein proteins have subunits that disassemble, change conformation, and reassemble into different quaternary structures. In some cases, these structural changes are involved in regulation of a single function, but in other cases, the structural changes are correlated with a switch between two different functions.

In this next section, we describe examples of IDPs, metamorphic proteins, and morpheeins and how variability in protein structure contributes to some of these proteins being moonlighting proteins with “fuzziness” in protein function.

#### 2.4.1. Intrinsically Disordered Proteins

Intrinsically disordered proteins contain regions, sometimes the entire polypeptide chain, that are unfolded under physiological conditions. Some unfolded regions are fully functional without becoming completely folded, and others undergo reversible folding when binding with other molecules [60,61]. In some cases, this flexibility enables one IDP to bind to a variety of cellular components, including small molecules, other proteins, DNA, or RNA, which enables many proteins to perform more than one function and contributes to the complexity in cellular metabolism and regulation of transcription and translation, molecular translocation, DNA repair and replication, and cell signaling [62,63,64,65].

Thirteen proteins in the database of moonlighting proteins (MoonProt, moonlightingproteins.org) are also found in the database of intrinsically disordered proteins (DisProt.org) (Table 1) [5,66]. Two proteins are discussed further below, p53 and thymosin beta-4.

The tumor suppressor protein p53 is a moonlighting protein and also an IDP with roles in cell cycle regulation, DNA repair and apoptosis [67,68]. In normal cells, the levels of p53 are low, but if cells sense environmental dangers that cause DNA damage, such as toxins, viruses or radiation, the level of p53 rises. Then, p53 binds to regulatory elements in the genome, activating a cascade of cellular responses to stop cell division and prevent cells from uncontrolled growth. In DNA repair, p53 can interact directly with DNA polymerase and AP endonuclease to stimulate base excision repair [69]. Outside the nucleus, p53 also has several cytoplasmic functions, including in centrosome duplication, induction of apoptosis and inhibition of autophagy [68].

In the native state, p53 has both folded and unfolded domains. The folded core domain is a DNA binding domain that recognizes specific regulatory elements. The folded tetramerization domain at the center of the protein joins protein subunits together into a homo-tetramer [70]. The *N*-terminal transactivation domain that interacts with and activates transcription factors is intrinsically disordered [71]. The *C*-terminal domain and the linker regions in between domains are also intrinsically disordered and fairly flexible, which enables the protein to adjust its conformation upon binding to specific regulatory sites in the DNA [72]. The flexibility of the intrinsically disordered domains allows p53 to recognize and bind to a large number of regulatory elements, so that it can regulate transcription in many different sites of the genome.

Thymosin beta-4 (Tβ4) is another moonlighting protein that is also an IDP. Tβ4 mainly functions in sequestering G-actin (monomeric actin) to prevent it from polymerization [73,74]. Tβ4 also has multiple moonlighting functions that are involved in diverse cellular roles including enhancement of endothelial cell differentiation, stimulation of angiogenesis, tissue regeneration and inhibition of inflammatory responses [75,76,77,78].

The free form of Tβ4 is intrinsically disordered and predominantly unstructured in solution. However, upon binding with G-actin, Tβ4 becomes fully folded and structured, where an extended conformation in the central region and two helices at the *N*-, *C*-termini can be identified [79,80]. In addition, Tβ4 forms complexes with PINCH (Particularly Interesting New Cys His-containing protein), ILK (Integrin-Linked Kinase) and stabilin-2 (an endocytic receptor for hyaluronic acid) respectively, where weak, transient and structurally ambiguous protein–protein interactions take place [81,82,83].

Protein structures in which significant conformational heterogeneity, disorder or ambiguity remain after formation of the complex are referred to as “fuzzy interactions” or “fuzzy complexes” and the remaining flexibility or disorder can be important in the assembly or activity of the complexes [116,117,118]. Fuzzy interactions and complexes can enable interactions with alternative partners and sensitivity to post-translational modifications. Fuzzy interactions also play a large part in several types of supramolecular interactions including in intracellular lipid droplets, which are described in another paper in this collection by Uversky [119].

The fuzzy complexes formed between IDPs and their binding partners, as mentioned above, can be an important feature of IDPs in fulfilling their functional versatility. Moonlighting proteins GCN4, HMGB1, CFTR, and Ure2 are found to be part of fuzzy complexes in the Fuzzy Complex Database (http://protdyn-database.org) [120]. As an example, GCN4 is a transcription activator for several genes, and is also a ribonuclease [86]. As a transcription activator, GCN4 binds Gal11 (an activator) in a weak and low affinity mode with multiple conformations. This conformational ambiguity is a typical example of a fuzzy complex where no single binding conformational state has been identified [117].

#### 2.4.2. Metamorphic Proteins

Metamorphic proteins add another layer of complexity to our understanding of protein structure and function [121,122]. In stark contrast to the dogma of one sequence, one structure, one function, metamorphic proteins have two or more folded structures as their native structures, and in some cases the different structures have different functions. Distinct from the intrinsically disordered proteins, where the native states are folded or unfolded, the native states of metamorphic proteins are both folded and structured. The interconversions between native structures are reversible, meaning that at equilibrium there is a balance between the native structures [123]. Although the fact of two native structures appears to be against the thermodynamic principles of protein folding, in which the native structure of a protein has the overall lowest free energy, it has been shown that the two native structures can have similar energies with a low activation barrier of refolding [124]. So far, a small number of metamorphic proteins have been discovered, including lymphotactin, RfaH, CLIC1, Mad2, KaiB, IscU, Selecase and HIV-1 reverse transcriptase, of which the first three are also moonlighting proteins [125,126,127,128,129,130,131,132].

**Figure 3:**
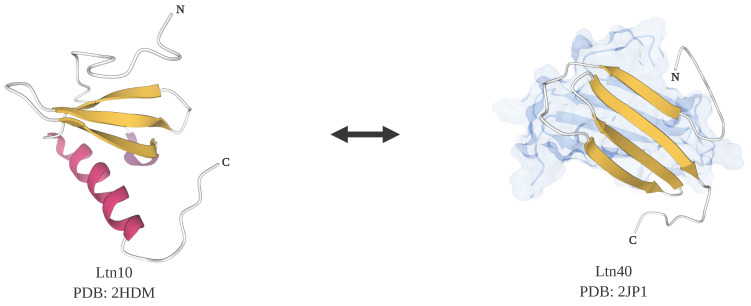
Structures of lymphotactin, a metamorphic protein that is also a moonlighting protein. There are two tertiary folds for lymphotactin, Ltn10 and Ltn40. Ltn10 has a classical chemokine fold with a mix of alpha-helix and beta-sheet (PDB ID: 2HDM [134]). Ltn40 possesses a dimeric form with each subunit composed of mainly beta-sheets (PDB ID: 2JP1 [125]). Note: This figure was “Created with BioRender.com”, and the visualizations of the protein structures were created with Mol* [36] on the RCSB PDB website (rcsb.org) [37,38].

The C family chemokine lymphotactin (Ltn) is a metamorphic protein and also a moonlighting protein with heparin-binding activity [133]. Under normal physiological conditions, 37 °C and 150 mM NaCl, lymphotactin exists in an equilibrium between two native states, Ltn10 and Ltn40. Ltn10 is a monomer possessing a mix of beta sheet and alpha helix in a canonical chemokine fold that undergoes refolding and dimerization to become Ltn40, which contains a beta sandwich (Figure 3). While Ltn10 is an agonist for the X-C G-protein coupled chemokine receptor 1 (XCR1), Ltn40 can’t bind to XCR1 but instead can bind to heparin, a glycosaminoglycan component of the extracellular matrix. At equilibrium, there are nearly equal amounts of Ltn10 and Ltn40. The interconversions between Ltn10 and Ltn40 can be controlled by small changes in salt concentration and temperature. When the salt concentration is high and the temperature is low, the presence of Ltn10 is predominant, however, at lower salt concentrations and higher temperatures, but still below 40 °C, Ltn40 is the predominant species [125]. Most other chemokines don’t appear to undergo these transformations because they contain two disulfide bonds. Because lymphotactin only has one disulfide bond, it is less restricted and more flexible in changing conformations compared to other chemokines, which partially explains the reversibility between two distinct native structures. In experiments where an extra disulfide bond was introduced, lymphotactin could be locked in only one native state and the transition to the other state was prohibited, suggesting that a single amino acid modification can change the functionality of lymphotactin significantly [134].

Another metamorphic protein with moonlighting activity is RfaH, which functions as a transcription factor that inhibits termination and is also a translation factor. RfaH has two domains: a *C*-terminal domain (CTD) and an *N*-terminal domain (NTD). As a transcription factor, these two domains are tightly bound together, and the CTD is in an all-helical conformation that masks the RNAP (RNA polymerase) binding surface on the NTD, preventing the NTD from interacting with RNAP. When the NTD binds to specific operons in DNA, the two domains are separated, which enables the NTD to interact with RNAP. Meanwhile, the CTD undergoes a transformation from an all-helical conformation to an all-beta one, after which the CTD is able to recruit ribosomes and potentiates the translation of operons controlled by RfaH [126,135].

In addition, CLIC1 is a metamorphic protein with two native folded states and also a moonlighting protein with two different functions [136]. In an oligomeric form, CLIC1 functions as a transmembrane chloride ion channel with its *N*-terminus folded in an all-alpha native state. In a monomeric and soluble form, CLIC1 functions as an oxidoreductase with a transformed *N*-terminus that is a mixed structure containing an alpha helix and a beta sheet [137,138].

#### 2.4.3. Morpheein Proteins

The subunits of morpheeins form a multimer that can disassemble, change conformation (without refolding), and reassemble into a different multimer [139].

**Figure 4:**
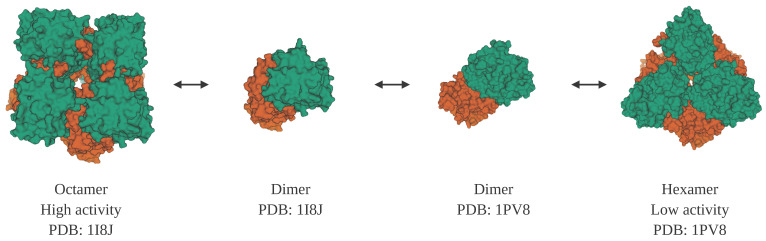
Structures of porphobilinogen synthase, which is both a morpheein and a moonlighting protein. Porphobilinogen synthase can form two homo-multimers, a low activity hexamer (PDB ID: 1PV8 [143]) and a high activity octamer (PDB ID: 1I8J [144]). The two multimers can interconvert through two homo-dimers, with different subunit conformations. In addition to its catalytic function, porphobilinogen synthase has a second function as an inhibitor of the proteasome. Note: This figure was “Created with BioRender.com”, and the visualizations of the protein structures were created with Mol* [36] on the RCSB PDB website (rcsb.org) [37,38].

Porphobilinogen synthase (also known as delta-aminolevulinic acid dehydratase) is the prototype of a morpheein. It has two oligomeric states that correlate with different levels of enzyme activity and binding of allosteric effectors [140]. An octamer can disassemble into dimers. While part of a dimer, domains within the subunits can shift in their relative positions to result in subunits with a different conformation that can then assemble into a hexamer (Figure 4). While these different homomultimers vary in their level of activity of one function, porphobilinogen synthase enzyme activity, the protein is also a moonlighting protein because it has a second function in which it binds to and inhibits the proteasome [141,142].

The ebolavirus VP40 is a morpheein and moonlighting protein that has three different functions in the virus life cycle, and each of these functions corresponds to a different arrangement of subunits [145]. An octameric ring structure binds to viral RNA to regulate its transcription while in host cells. A butterfly-shaped dimer moves to the host cell’s plasma membrane. Then a linear hexameric form assembles into a larger structure that is needed for budding.

## 3. Moonlighting Proteins in Cellular Complexity

The variety of functions and combinations of functions of moonlighting proteins contribute to the complexity of cellular metabolism. Protein function, and in many cases the structure, is dependent on cellular factors that can vary due to intracellular conditions and the extracellular environment, and the output can change in a dynamic way. The examples given above are just some of the known factors affecting the functions of moonlighting proteins, and one protein often responds to multiple signals or combinations of signals. Some switches in function are reversible, others are not. Within a single cell, some copies of a moonlighting protein can be performing one function, some another, and some both simultaneously, depending on the protein, the cell type, and on the individual cell’s metabolic state and environmental conditions.

These factors that make our understanding of the cell difficult are valuable to the cell because they help enable dynamic responses to fluctuations in conditions within the cell and in its environment. Because the function of moonlighting proteins can depend on multiple factors, they can also be components of controllable cellular responses and be involved in processing information. Some moonlighting proteins also help regulate the level of activity of other proteins in the cell, for example by an enzyme with a second function in a cell signaling pathway or as translation or transcription factors. Because moonlighting proteins can have different activities in different cell types, they also contribute to different cell types having different phenotypes with specialized functions.

In addition to actually contributing to the complexity of the many interconnected pathways and processes in the cell, the ability of a protein to perform two different functions adds to the “fuzziness” of our inability to fully understand, predict, and model the activities in a cell and how they interact and are regulated. There are many things we still don’t know about moonlighting proteins. First, we don’t know how many proteins are moonlighting proteins. Many protein functions were found by serendipity and researchers are often looking for one type of function when they study a protein, not all of the functions that might be there. There are also many proteins identified through sequencing projects for which we don’t know any functions. We also don’t completely understand the triggers and mechanisms for switching between different functions—the cellular conditions, ligands, protein–protein interactions, conformational changes, PTMs, etc. involved and how all the different triggers combine.

Our understanding of moonlighting proteins and our ability to predict which proteins are moonlighting proteins and what are their functions is also complicated because a protein can have one second function, but a homologous protein can have a different second function, for example the cytoplasmic and mitochondrial aconitases mentioned above. Leucyl-tRNA synthetase is another example. It is an enzyme that attaches leucine to tRNA, but it has additional functions that vary in yeast and humans. In the yeast *Saccharomyces cerevisiae*, leucyl-tRNA synthetase is involved in intron splicing in RNA [146]. However, in humans it is involved in cell signaling, where it senses the cellular leucine concentration and binds to and activates Rag GTPase, leading to the activation of mTORC1 (mammalian target of rapamycin complex 1) [147].

In fact, a family of homologous enzymes can include enzymes with the canonical catalytic function of the protein family, moonlighting proteins with different combinations of catalytic and non-catalytic functions, as well as enzymes with variations on the canonical catalytic function (i.e., different substrates or chemical reaction catalyzed) and even pseudoenzymes, which can resemble active enzymes but have no catalytic function [148,149]. Though noncatalytic, pseudoenzymes play important roles in regulating the activity of their catalytic homologues, facilitating the assembly of scaffolding complexes and coordinating transcription and translation [150]. The loss of the catalytic functions can be attributed to a variety of aspects, such as the loss of essential amino acid residues needed for catalysis in the active site, a blockage of the entrance to the active site, and mutations of the amino acids involved in binding the substrate [151,152]. The first reported pseudoenzyme was alpha-lactalbumin, which is homologous to the enzyme lysozyme but does not have catalytic activity [153,154]. Instead, alpha-lactalbumin is a component of lactose synthase, serving as a regulatory subunit that increases the substrate binding affinity for the catalytic subunit of the enzyme [155]. Another example is found in the argininosuccinate lyase protein family. The canonical argininosuccinate lyase enzymes catalyze the breakdown of argininosuccinate into arginine and fumarate. Delta1-crystallin is a pseudoenzyme member of the family with a function as a structural protein in the lens of the eye in birds and reptiles. Another member of the protein family, delta2-crystallin, shares 94% amino acid sequence identity with the delta1-crystallin and is both a crystallin and a catalytically active argininosuccinate lyase that catalyzes the breakdown of argininosuccinate into arginine and fumarate [156,157].

## 4. Conclusions

The variability in the functions of moonlighting proteins, including some intrinsically disordered, metamorphic and morpheein proteins, contribute immensely to the fuzziness concept of cellular metabolism as described by Gentili [6]. Many types of proteins have multiple functions that interact in a complex pattern of interacting pathways and processes. As cellular conditions change due to metabolism and environmental conditions, the functions of these proteins change, resulting in different combinations of interactions and processes. The fuzziness concept also represents our limited understanding of these players and our inability to fully predict and model their actions and interactions. We don’t yet know how many proteins are moonlighting proteins or the full complement of their functions, and in many cases, we also don’t know the cellular factors that affect their functions. Moreover, while many advances have been made in our ability to predict protein functions, the variety of functions found even among homologous proteins adds to the fuzziness of our predictions.

## Figures and Tables

**Table 1:** Examples of moonlighting proteins that contain intrinsically disordered regions.

**Protein**	**Organism**	**Function 1**	**Function 1 Location**	**Function 2**	**Function 2 Location**	**Disorder Content**	**UniProt ID**	**Ref.**
P53	*Homo sapiens*	Binds to regulatory element in the genome	Nucleus	Centrosome duplication, induction of apoptosis, autophagy inhibition	Cytoplasm	37.00%	P04637	[67,69,71]
Thymosin beta-4	*Homo sapiens*	Involved in sequestering G-actin in human polymorphonuclear leukocytes	Cytoplasm	Secreted anti-inflammatory agent	Extracellular	100.00%	P62328	[73,78,84]
General control protein GCN4	*Saccharomyces cerevisiae*	Transcription factor	Nucleus	Ribonuclease	Cytoplasm	32.38%	P03069	[85,86,87]
Bifunctional ligase/repressor BirA	*Escherichia coli*	Biotin synthetase, biotin–[acetyl–CoA-carboxylase] ligase	Cytoplasm	Biotin operon repressor, activity depends on cellular concentration of biotin	Bound to DNA	7.17%	P06709	[42,43,88,89]
High mobility group protein B1	*Rattus norvegicus*	Binds heparin.	Cytoplasm	DNA binding protein, without sequence specificity	Nucleus	35.81%	P63159	[90,91,92]
Calreticulin	*Homo sapiens*	Protein-folding chaperone	Endoplasmic reticulum	Adhesin	Cell surface	100.00%	P27797	[93,94,95]
50S ribosomal protein L4	*Escherichia coli*	Ribosomal protein, part of the 50S subunit	Cytoplasm	Transcriptional repressor, causes premature termination of transcription	Bound to DNA	31.34%	P60723	[48,96,97]
DNA replication factor Cdt1	*Homo sapiens*	Helps with initiating DNA replication.	Nucleus	Role in mitosis, localizes to kinetochores through binding to Ndc80 complex	Cytoplasm	2.20%	Q9H211	[98,99,100]
Transcriptional regulator Ure2	*Saccharomyce cerevisiae*	Binds to and inhibits GATA transcriptional activators GLN3 and GAT1	Cytoplasm	Glutathione peroxidase, thiol:disulfide oxidoreductase	Cytoplasm	31.64%	P23202	[101,102,103]
Thymidylate synthase	*Homo sapiens*	Thymidylate synthase	Cytoplasm	mRNA translation inhibition	Cytoplasm	15.65%	P04818	[104,105,106]
Cytochrome c	*Equus caballus*	Electron carrier protein	Mitochondrion	Binds to apoptosis protease activation factor-1 and promotes apoptosis	Cytoplasm	99.05%	P00004	[107,108,109]
Band 3 anion transport protein	*Homo sapiens*	Transports inorganic anions across the plasma membrane	Plasma membrane	Scaffold protein providing binding sites for glycolytic enzymes	Plasma membrane	5.93%	P02730	[110,111,112]
Cystic fibrosis transmembrane conductance regulator CFTR	*Homo sapiens*	Chloride transporter, contains nucleotide binding domains that bind and hydrolyze ATP	Plasma membrane	Regulator of other ion channels	Plasma membrane	12.50%	P13569	[113,114,115]
